# CD63 and GLUT-1 Overexpression Could Predict a Poor Clinical Outcome in GIST: A Study of 54 Cases with Follow-Up

**DOI:** 10.1155/2016/6478374

**Published:** 2016-10-04

**Authors:** Piotr Lewitowicz, Jarosław Matykiewicz, Dorota Koziel, Magdalena Chrapek, Agata Horecka-Lewitowicz, Stanislaw Gluszek

**Affiliations:** ^1^Department of Pathology, Faculty of Medicine and Health Sciences, Jan Kochanowski University in Kielce, Kielce, Poland; ^2^Department of Surgery and Surgical Nursing, Faculty of Medicine and Health Sciences, Jan Kochanowski University in Kielce, Kielce, Poland; ^3^Department of General, Oncological and Endocrine Surgery, The Voivodeship Hospital in Kielce, Kielce, Poland; ^4^Department of Probability Theory and Statistics, Institute of Mathematics, The Faculty of Mathematics and Natural Sciences, Jan Kochanowski University in Kielce, Kielce, Poland; ^5^Department of Public Health, Faculty of Medicine and Health Sciences, Jan Kochanowski University in Kielce, Kielce, Poland

## Abstract

*Background and Goals.* In light of current knowledge, it seems that alternations underlying GISTs are well explained, although all that is enhanced by various aspects on a daily basis. More recently, attention has been pointed towards exosomes as important particles able to modify healthy and also diseased tissues including cancer. The goal of the present study was an analysis of CD9, CD63, and GLUT-1 as a marker of hypoxia status within 54 cases of GIST and evaluation of their predictive value.* Methods.* 54 cases of patients suffering from GIST were enrolled into the study, predominantly in the gastric location. All operated cases had no Imatinib and other chemotherapies up to the day of operation. Expression of targeted proteins was performed by immunohistochemistry and, after that, the results with tabulated clinical data were compared by Kaplan-Meier method and multivariate Cox proportional hazard model of statistical analysis.* Results.* Our results presented a marked dependence of worsening clinical outcome with high expression CD63 (*p* = 0.008) as well as with GLUT-1 (*p* = 0.014). We noted a strict correlation of GLUT-1 expression with CD63 expression (*p* = 0.03), which could confirm the thesis about the contribution of exosomes in intratumoural hypoxia status. The collected material did not confirm CD9 contribution.* Conclusions.* As presented here, CD63 and GLUT-1 have a prognostic value in GIST cases. The results confirm the other studies in this scope and can be used in future as an additional prognostic factor.

## 1. Introduction

*Historical Background.* The understanding of molecular alternations underlying GIST began with the publication of Hirota et al. in 1998 [1]. The authors, first, had presented c-kit mutation within GIST, derived from pacemaker Cajal cells, and after that breakthrough GIST has been recognised as unique entity. The following problem was categorisation of biological potential. Having been created, canonical rules have been concerned with* tumour size*,* mitotic activity*, and* tumour location* and, what is important, they have been little transformed for many years. A huge positive quality of the current classification is its simplicity. To date, they remain very conservative although a few performed changes [2, 3]. The progress of available diagnostic methods has greatly simplified a preoperative tumour diagnosis in the recent past. Although the small biopsy material is often sufficient to state the GIST diagnosis, preoperative patients' management might be improved by knowing the additional features predicting the tumour's aggressive potential. In these cases, the key prognostic factor such as* mitotic activity* is obviously valueless and prediction based only on tumour measurement and location could be supported by other ones.

*Searching for Other Targets and Predictive Factors.* Many studies have been performed towards searching of the cancer's targets. Imatinib was used for the treatment of GIST after discovering the c-kit mutation and thereby became the paradigm of molecularly targeted personalised therapies. Contrary to growth-factors-linked targeting therapy, neoangiogenesis and extracellular environment modelling factors were widely investigated but in fact the palpable benefits for the treatment remain restricted. With regard to the unquestionable tumour heterogeneity, where various pathways are often involved, there is an obvious need of seeking others. Recently, an attention has been clearly drawn also to exosomes as the potential target in personalised treatment.

Exosomes are bilayer cell-membrane bound nanovesicles which are stored and released to an extracellular environment where they can modulate numerous pathways. They exist in both normal and neoplastic cells and their transmembrane 4 superfamily proteins (TM4SFP) are called tetraspanins. CD9, CD63, CD81, and CD151 are included in this family as the most important proteins [4]. They usually exist in the vascular system where their contribution to endothelial proliferation, neovascularisation, smooth muscle fiber motility, and contraction is clearly evident. There are evidences of their potent share in downregulation of intratumoural immune host response, remodelling of extracellular environment, especially with MMP-1, chemoresistance, epithelial-mesenchymal transition, angiogenesis, metastasis, intracellular communication with trafficking, and, additionally, various nanoparticles, often containing genetic material like mRNA, microRNA, long noncoding RNA, or DNA [5, 6]. Obviously, these characteristics have a very harmful action in the cases of cancer. Moreover, very important observation has been noted about synergy of c-kit with CD9 as well as CD63. Namely, c-kit as a transmembrane cytokine receptor might exhibit various phenotypes secondary to binding with tetraspanins proteins. Binding of TM4SF complex to c-kit could result in basal phosphorylation of c-kit and paradoxically reduces kinase activity and downstream with proproliferating effect [7]. That observation has not been fully elucidated but it is very interesting in light of cases of chemoresistance to Imatinib especially with secondary c-kit overexpression and loss of tetraspanins. All this could modify the clinical outcome and impact the choice of first-line treatment in the future. Additionally, it is highlighted that intratumoural hypoxia can induce, via tumour-derived exosomes, angiogenesis and supplies oxygen and nutrients to cancer [5]. Because of an explicit rise of Imatinib chemoresistance, usually after two years of therapy, various studies are trying to explain pathomechanism involved. It seems that second, acquired, KIT mutation is mainly responsible for c-kit overexpression but, on the other hand, chemoresistance might result also from the changes of tetraspanins expression [8–10]. The current opinion about the contribution of exosomes in chemoresistance is not clear. To date, a few trials have been executed with studying of exosomes (https://clinicaltrials.gov/) but most are at the stage of subject recruitment.

GLUT-1 is an uniport transmembrane protein that facilitated an insulin-independent intracellular glucose transport. It has a wide distribution among various tissues but actually interesting is the fact of its overexpression within hypoxic tissues especially cancers. Although the mechanism leading to that is not completely clear, the exosomes' contribution is taken into consideration.

The abovementioned theses indicate tetraspanin family proteins as additional important prognostic factors; thus, further studies with clinical material are required.

## 2. Aims

In order to enhance the tumour's characteristics, we were looking for dependency between intratumoural hypoxia level, exosomes, and clinical outcome. We aimed to study intratumoural hypoxia status using GLUT-1 and CD9 and CD63 as tetraspanin family proteins.

## 3. Materials and Methods

Fifty-four (54) cases of CD117 positive GISTs cases operated on at the Clinical Department of Surgery in Kielce, Poland, headed by Professor S. Gluszek, M.D., over the period of 1998–2015 have been enrolled into the study. The collective data for evaluation of follow-up were tabulated and enhanced annually in most of the cases. A computer tomography examination of the abdomen and chest, a typical blood test, and an endoscopic examination were performed. Each case was rediagnosed according to the recent ESMO classification based on model Miettinen's classification [2, 11]. Importantly, all the studied participants underwent typical surgical treatment solutions without previous treatment of Imatinib and other chemotherapies. This resulted in a credible comparative analysis of tumour characteristics in the scope of treatment, unchanged expression of the searched proteins. An achievement of the goals was performed by the examination of GLUT-1 (ready to use solution, number 760-4526; rabbit polyclonal, Cell Marque), CD9 (CD9: EPR2949, Biogenex, diluted at 1 : 30), and CD63 (ready to use solution; NKI/C3, Cell Marque) by immunohistochemical procedures with the use of automated IHC/ISH slide staining system BenchMark XT (Ventana Medical Systems; Roche Group, Tucson, USA). After deparaffinisation and rehydration of the samples, the unmasking processes (CC1), incubation with primary antibodies (time and temperature of incubation were in line with manufacturer's recommendations), and the further routine steps were performed. As secondary antibody we used the Ventana ultra View Universal DAB Detection Kit (Ventana Medical Systems; Roche Group, Tucson, USA). For positive control, we used renal cell carcinoma tissue for CD9 and malignant melanoma tissue for CD63 in concordance with the manufacturer's recommendations. Red blood cells were an internal positive control for GLUT-1. The value of CD9, CD63, and GLUT-1 was evaluated routinely for immunohistochemistry method as follows:* 0, lack; 1, weak; 2, moderate; and 3, strong expression.* The reached expression levels of CD9, CD63, and GLUT-1 were tabulated as 0-1 as low expression but 2-3 as high expression. Cut-off criteria of overexpression were stated as moderate or strong expression with a wide distribution. Weak expression and/or patchy reactions were classified as negative.

This study with the use of human tissues was in concordance with the ethical standards of the Declaration of Helsinki with its latest revision in 2004. Additionally, the study was approved by the Ethical Commission of the Faculty of the Medicine and Health Science, the Jan Kochanowski University in Kielce, Poland.

## 4. Statistical Methods

Quantitative data were reported as mean and standard deviation or median and range. Categorical data were expressed as number and percentage distributions. Chi-square test or Fisher's exact test was applied to compare proportions, and multivariable logistic regression model was used for assessing the relationship between the GLUT-1, CD63, and ESMO group. Follow-up time was calculated as the number of years from the date of surgery to disease recurrence, death from reasons other than GIST, or the last contact with the patient. For analysis of disease-free survival, deaths which were not related to GIST were treated as censored observations. Disease-free survival rates were estimated by Kaplan-Meier method. The univariate associations between disease-free survival and selected patient's and tumour's characteristics were evaluated by univariate Cox proportional hazard model, and for those analyses continuous variables were dichotomised according to median. To identify the independent prognostic factor for disease-free survival, multivariate Cox proportional hazard model with backward selection (with cut-off 0.15), performed on only those variables which were statistically significant in univariate analysis, was done. Hazard ratios in univariate and multivariate Cox model were estimated with 95% confidence intervals.

All statistical tests were two-sided and *p* values < 0.05 were considered significant. Computations were performed using STATISTICA (data analysis software system), StatSoft, Inc. (2014), version 12, http://www.statsoft.com/.

## 5. Results

At time of diagnosis, the average age of patients was 62.6 years and there were no differences according to gender. The most patients (68.6%) were in the 5th to 7th decade of their life, and the stomach was the most frequent tumour location (74%). The patients' division depending on tumour measure and ESMO group is presented in Table 1.

ESMO groups and GLUT-1, as well as ESMO groups and CD63, were significantly dependent. Namely, lack or weak expression of GLUT-1 positively correlated with 1st-2nd level of ESMO groups in comparison with 3rd or above level of ESMO groups (*p* value = 0.014 [chi-square test]). Similarly, low and weak expression of CD63 was noted in 1st-2nd ESMO groups in comparison with 3rd or above ESMO groups (*p* value = 0.003 [chi-square test]). There was no statistical significant dependence between ESMO groups and CD9 (*p* value = 0.25, exact Fisher's test). To summarise the above, low CD63 and GLUT-1 expression were connected with the first and second ESMO group in comparison to high level CD63 and GLUT-1 expressed within the third and above ESMO groups. These data suggest improving expression of these proteins with progress of tumour' aggressiveness level.

Moderate and strong expression of CD9 were presented in 98% of cases. As it is depicted in Table 2, all cases with poor clinical outcome showed strong expression and, in addition, only one case presented a lack of CD9.

The correct statistical analysis failed in this area due to these characteristics. As with the result above, the analysis of intratumoural hypoxia status with CD9 and CD63 was restricted only to CD63. Weak GLUT-1 expression positively correlated with weak CD63 expression in comparison to moderate and strong CD63 expression (*p* value = 0.03 [chi-square test]); however, the addition of ESMO groups in the logistic regression model showed a lack of correlation (*p* = 0.26, logistic regression model).

**Figure 1:**
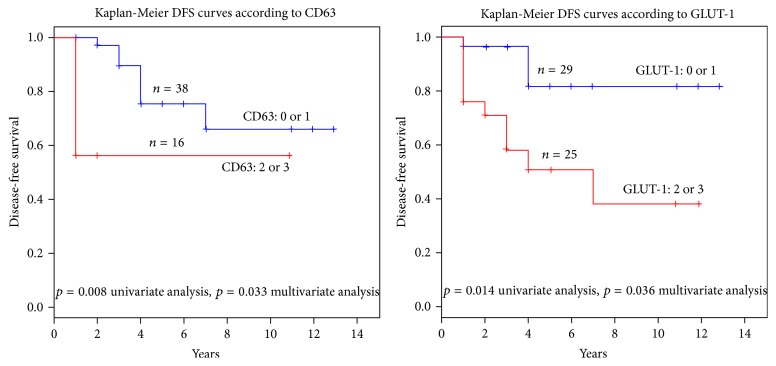


**Figure 2:**
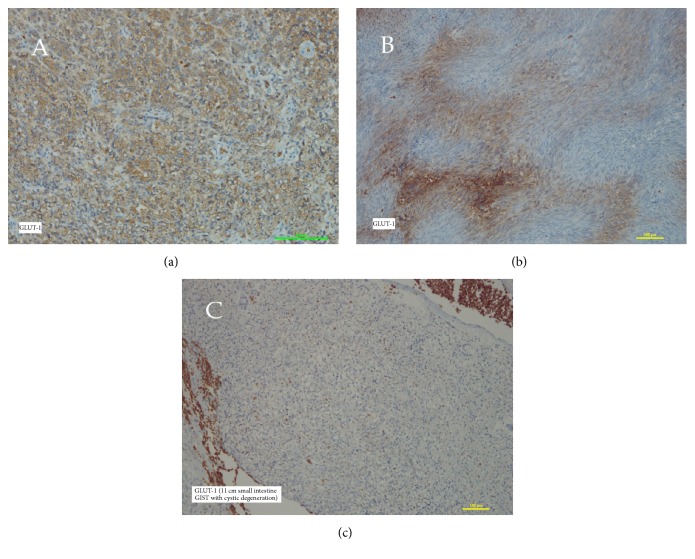


**Figure 3:**
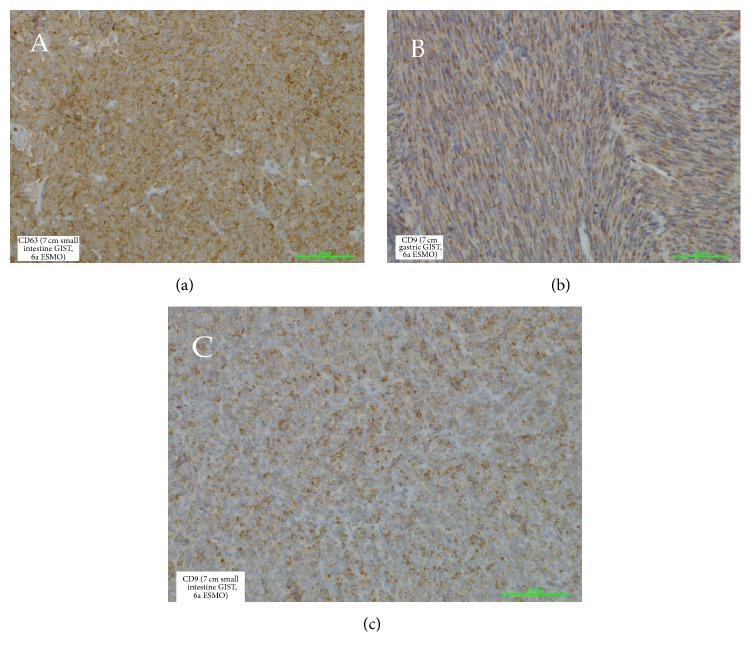


The main target of our study was the analysis of the utility of the studied proteins in connection with helping to predict the patient's survival. Not surprisingly, we observed the dependence of the risk of tumour recurrence with tumour measurement (*p* = 0.039) and ESMO group (*p* = 0.043). We have intentionally not presented here a correlation with mitotic activity due to a parallel conducted study in the scope of mitotic activity and cell-cycle connected proteins. The differences between gastric and nongastric located tumours in the range of recurrence risk have not been presented (*p* = 0.31). Obviously, this contradiction with well-established large cohort data results from the numerally limited studied group. Indeed, the most important result of the current study is the high risk of recurrence with strong expression of GLUT-1 and CD63. These data showed the strong correlation between expression of CD63 and disease-free period (*p* = 0.008 and *p* = 0.033 in a univariate and multivariate model, resp.). Similar observations concerned GLUT-1 (*p* = 0.014 and *p* = 0.036 in a univariate and multivariate model, resp.) (Table 2). As presented in Figure 1, the Kaplan-Meier curves clearly show a breakdown of the survival rate in the dependence of high expression of both CD63 and GLUT-1.  Figure 2 depicts various patterns of GLUT-1 expression and interestingly a lack of GLUT-1 in 11 cm small intestine tumour. Our observations have not indicated differences in expression targeted proteins in comparison to tumour location (*p* = 0.31) what is partially presented in Figure 3.

## 6. Discussion

The potent contribution of extracellular microenvironment in cancer progress is beyond question. The meaning of angiogenesis, extracellular environment remodelling, invasion, and metastases is mainly analysed in the scope of various growth factors, enzymes, cytokines, and so forth. In fact, it seems that the contribution of exosomes is no less important. Very often, they deliver mRNA, microRNA, or oncogenic signaling proteins which are spread around in the extracellular environment afterwards. Consequently, they are absorbed by neighbouring cells and begin a specific pathway of intercellular communication [4, 12–16]. To make things worse, the same occurs in cancers where they could play a leading role in tumor's growth. A usual phenomenon such as intratumoural hypoxia could, via excessive shedding of exosomes, induce angiogenesis and in fact a better blood supply [16]. That is in line with observations of strong expression of GLUT-family proteins in various cancers with high grade malignancy and has an obvious predictive value [17–19]. Our results have shown the strong dependence of CD63 expression with GLUT-1, which confirms the thesis of hypoxia-induced exosomes releasing. We have presented a collapse of survival-time-period in GISTs with high level of GLUT-1 expression. Moreover, it is logical that high expression GLUT-1 was closely connected with the third and above ESMO group. It proves, similarly as in various epithelial malignancies, that the progress of intratumoural hypoxia within GIST is secondary to the progress of its malignancy potential.

Similarly to GLUT-1, we have noted a significant correlation between poor clinical outcome and high CD63 expression. In our opinion, these observations, being in agreement with other studies, make these parameters valuable for the prediction of tumour behaviour. The progress of the malignant potential of GIST, connected with improving CD63 expression, is very thought-provoking, especially in light of the observations of a decline in the CD9 expression when tumours are getting more aggressive. In our opinion, it could be linked with their construction and intracellular location. The CD9 function is connected with the cell membrane and plays a multidirectional activity in the stabilisation of other membrane-bound proteins and regulation of the various “gates” into the cell. CD63 particles are both cell-membrane bound and separately floating into cytoplasm delivering various nanoparticles. Our result based on 54 tumours has shown above 98% moderate to strong expression of CD9. It does not confirm other results especially Yang' and Setoguchi' [20, 21]. The latter mentioned authors' researches have been very similar to ours in the range of CD9 IHC methodology, although they studied more numerous groups—we suppose that it and subjective attempt to splitting* low and moderate* expression might be a reason of discrepancy. Moreover, the very interesting work of Setoguchi et al. has also indicated the predictive value of the* versican* protein and the authors noted marked GIST heterogeneity by molecular analysis of both primary and liver-metastatic tissues. The results about CD9 meaning in GIST are very few in the literature, and, in fact, they are restricted to Yang's and Setoguchi's works. Much more has been evaluated in this field among various epithelial cancers where CD9 decline was connected with high grade cancers and worse clinical outcome [22–29]. It is possible that CD9 strictly cooperates and mirrors the expression of c-kit. The described fading CD9 with progress of GISTs' malignancy needs further studies.

Another very important factor connected with worse clinical outcomes is tumour fragmentation or rupture. For many years, clinical observations have provided the evidences of multivisceral tumour implementation after tumour rupture [30–32]. Idiopathic tumour rupture could be also partially a consequence of tetraspanins action. We remember their multidirectional operation, for example, cell motility, contraction, neovascularisation, vascular permeability, prothrombotic activity, and direct impact on secretion of metalloproteinases. All of these features contribute to tumour growth and intratumoural degenerative changes with consequently possible rupture. Indeed, there are GIST cases with massive stromal edema or cystic degeneration which obviously distort an actual tumour's measurement as well as the mitotic activity index.

## 7. Conclusions

In our opinion, it is very important to draw attention to the recently published additional pathways, often connected with tumour metabolism, that lead to cancer progression. Our results with GLUT-1 and CD63 confirm the results of other researches in the scope of various malignancies and should result in practical use in the future.

## Figures and Tables

**Table 1:** Characteristics of patients and tumours.

	General characteristics			CD9	CD63	GLUT-1
Age at diagnosis (years)	Mean (SD)	62.6 (13.6)		n/a		
	Median (min–max)	64.5 (31–89)				

		*n*	%			

Age at diagnosis (years)	30–39	2	3.7	n/a		
	40–49	9	16.7			
	50–59	13	24.1			
	60–69	11	20.4			
	70–79	13	24.1			
	80–89	6	11.1			

Gender	Female	28	51.9	n/a		
	Male	26	48.1			

Tumour location	Stomach	40	74.1	n/a		
	Small intestine	10	18.5			
	Colon	2	3.7			
	Duodenum	1	1.9			
	Rectum	1	1.9			

Tumour size (cm)	Median (min–max)	5	(0.8–23)	n/a		

Tumour size (cm)	≤2.5	11	20.4	n/a		
	2.51–5.0	20	37.0			
	5.01–7.5	12	22.2			
	7.51–10	3	5.6			
	>10	8	14.8			

ESMO group	1	6	11.1	ESMO 1-2 in comparison with 3a and above *p* = 0.25	ESMO 1-2 in comparison with 3a and above *p* = 0.003 /chi-square test/	ESMO 1-2 in comparison with 3a and above *p* = 0.014 /chi-square test/
	2	21	38.9			
	3a	10	18.5			
	3b	4	7.4			
	4	2	3.7			
	5	2	3.7			
	6a	6	11.1			
	6b	3	5.6			

CD9 expression	0	1	1.9	—	*p* > 0.05	*p* > 0.05
	2	7	13.0			
	3	46	85.2			

CD63 expression	0	21	38.9	*p* > 0.05	—	Comparison of CD63 0-1 with GLUT-1 0-1 and 2-3 expression *p* = 0.003 /chi-square test/
	1	17	31.5			
	2	9	16.7			
	3	7	13.0			

GLUT-1 expression	0	6	11.1	*p* > 0.05	Comparison of CD63 0-1 with GLUT-1 0-1 and 2-3 expression *p* = 0.003 /chi-square test/	—
	1	23	42.6			
	2	16	29.6			
	3	9	16.7			

Follow-up (years)	Median (min–max)	3	(1–13)	n/a		

n/a: not applicable.

**Table 2:** Univariate and multivariate Cox regression model for the recurrence of GIST.

Variable		Number of patients		Disease-free survival (%)		Univariate		Multivariate	
		Total	With recurrence	1-year	3-year	HR (95% CI)	*p* value		
Age (years)	≤64	27	5	96.3	86.5	1 (ref. group)	0.15		
	>64	27	9	77.8	70.7	2.24 (0.75–6.74)			

Sex	Female	28	8	92.9	76.0	1.42 (0.48–4.16)	0.53		
	Male	26	6	80.8	80.8	1 (ref. group)			

Tumour size (cm)	≤5	31	5	96.8	93.1	1 (ref. group)	0.039		
	>5	23	9	73.9	57.5	3.19 (1.06–9.61)			

Tumour location	Gastric	40	8	87.5	78.3	1 (ref. group)	0.31		
	Non-gastric	14	6	85.7	77.1	1.73 (0.60–5.04)			

ESMO group	1 or 2	27	4	100.0	95.8	1 (ref. group)	0.043		
	3 or more	27	10	74.1	60.6	3.34 (1.04–10.76)			

CD63	0-1	38	7	100.0	89.6	1 (ref. group)	0.008		
	2-3	16	7	56.3	56.3	4.52 (1.49–13.73)		3.48 (1.10–10.96)	0.033

GLUT-1	0-1	29	3	96.6	96.7	1 (ref. group)	0.014	1 (ref. group)	
	2-3	25	11	76.0	58.0	5.00 (1.39–17.96)		4.05 (1.10–14.89)	0.036

CD9	0–2	8	0	100.0	—	—	—		
	3	46	14	84.8	74.9	—			

## Additional Points

*Limitations of the Study.* The evaluation has been performed on ethnically homogenous Caucasian race people group. The fifty-four-item group as a cohort is relatively small in statistical evaluation. The immunohistochemical cut-off criteria for moderate and strong expression are often subjective and may result in statistical results. A predominance of gastric located tumours might be connected with achieved results.
